# Hepatic Sclerosing Hemangioma with Predominance of the Sclerosed Area Mimicking a Biliary Cystadenocarcinoma

**DOI:** 10.1155/2018/7353170

**Published:** 2018-10-04

**Authors:** Hiroyuki Sugo, Yuki Sekine, Shozo Miyano, Ikuo Watanobe, Michio Machida, Kuniaki Kojima, Hironao Okubo, Ayako Ura, Kanako Ogura, Toshiharu Matsumoto

**Affiliations:** ^1^Department of General Surgery, Juntendo University Nerima Hospital, Japan; ^2^Department of Gastroenterology, Juntendo University Nerima Hospital, Japan; ^3^Department of Diagnostic Pathology, Juntendo University Nerima Hospital, Japan

## Abstract

We report here an extremely rare case of hepatic sclerosing hemangioma mimicking a biliary cystadenocarcinoma. A previously healthy 39-year-old woman was referred to our hospital because of a large tumor in the liver. Abdominal computed tomography revealed early peripheral ring enhancement in the arterial phase and slight internal heterogeneous enhancement in the delayed phase. Magnetic resonance imaging revealed a tumor with low intensity in the T1-weighted image and very high intensity in the fat-saturated T2-weighted image. The patient underwent hepatectomy for a possible malignant liver tumor. Grossly, the tumor appeared as a white, solid, and cystic mass (weighted 1.1 kg and measured 170×100×80 mm) that was elastic, soft, and homogeneous with a yellowish area. Histological examination showed that the tumor mostly consisted of fibrotic areas with hyalinization. The typical histology of cavernous hemangioma was confirmed in part, and the tumor was diagnosed as a sclerosing hemangioma with predominancy of the sclerosed area. A review of 20 cases reported previously revealed that only 2 (10%) patients were diagnosed as having sclerosing hemangioma preoperatively.

## 1. Introduction

Hemangioma is the most common type of benign hepatic tumor [1]. Hemangioma degeneration can occur through an increase in the degree of fibrosis and thrombosis of its vascular channels, a condition known as sclerosing and/or hyalinizing hemangioma [2]. This can then lead to the end stage, known as the involution stage, in which the hemangioma becomes completely sclerosed and/or hyalinized [3, 4]. Sclerosing hemangioma is an extremely rare type of benign hepatic tumor, which mimics hepatic malignancies such as metastatic liver tumor or cholangiocarcinoma [5, 6]. We present herein a case of sclerosing hemangioma in a 39-year-old woman and review the relevant literature, with special reference to pathological features.

## 2. Case Presentation

**Figure 1:**
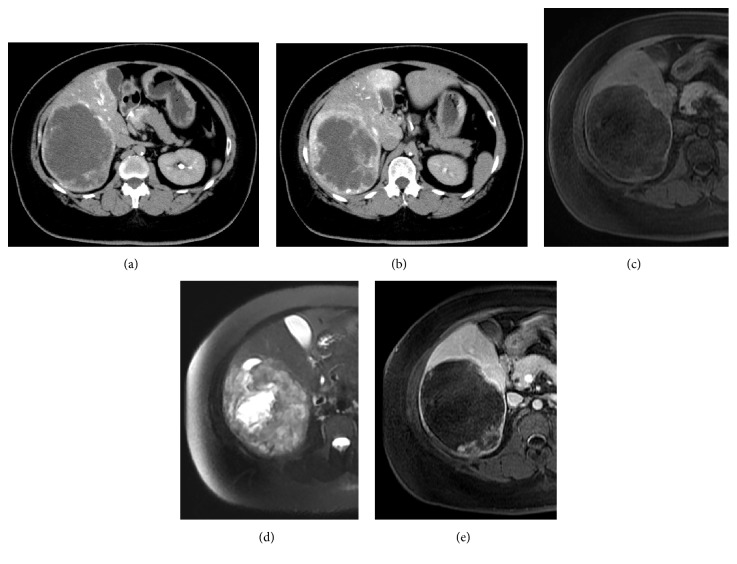
**Abdominal computed tomography during hepatic angiography and magnetic resonance imaging.** Arterial phase CT scan shows a geographic lesion in the right lobe of the liver with a rim and nodular enhancement (a), and the delayed phase of CT reveals heterogeneous enhancement in the peripheral area of the mass with a gradual centripetal enhancement pattern (b). The tumor shows low signal intensity on T1-weighted images (c) and some high-signal intensity nodules on T2-weighted images (d). EOB-MRI shows no uptake in the corresponding area (e).

**Figure 2:**
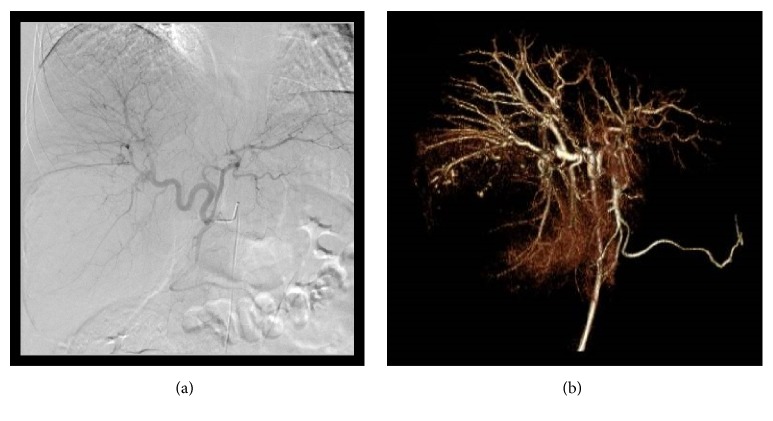
**Abdominal angiography.** (a) Common hepatic angiography image. (b) Three-dimensional image obtained by common hepatic angiography. Hepatic angiography shows a large avascular region in the liver corresponding to the tumor.

A previously healthy 39-year-old woman was referred to our hospital because of a cystic lesion in the liver demonstrated by abdominal ultrasonography (US). Laboratory studies, including liver function tests, and tumor markers were also within the normal limits. Serological markers for hepatitis B or C viral infection were undetectable. Abdominal US revealed a well demarcated, heterogeneously low-echoic mass 170 mm in diameter in right lobe of the liver. Abdominal computed tomography (CT) during hepatic arteriography (CTHA) revealed early ring enhancement in the peripheral area in the arterial phase and slight internal heterogeneous enhancement in the delayed phase (Figures 1(a) and 1(b)). Magnetic resonance imaging (MRI) showed that the tumor had low signal intensity on T1-weighted images and some foci of high signal intensity on T2-weighted images. Gadolinium ethoxybenzyl (Gd-EOB) MRI revealed no uptake in the corresponding area (Figures 1(c), 1(d), and 1(e)). Abdominal angiography demonstrated a large avascular region in the liver corresponding to the tumor, although no typical features of cavernous hemangioma were evident (Figure 2). 18-Fluorodeoxyglucose positron emission tomography (FDG-PET) revealed no abnormal FDG uptake. With these radiological findings, malignant liver tumor could not be excluded, such as biliary cystadenocarcinoma, cholangiocarcinoma, mesenchymal tumors, and hepatocellular carcinoma associated with cystic formation.

**Figure 3:**
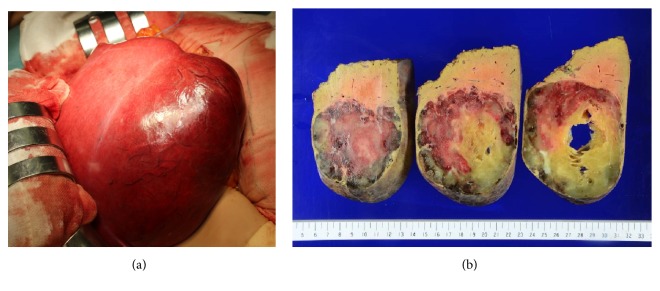
**Intraoperative findings and macroscopic findings of the resected tumor.** Exploration of the abdominal cavity showed a relatively soft, dark red tumor (a). The cut surface demonstrated a white solid and cystic mass (170×100×80 mm in size) that was elastic, soft, and homogeneous with multiple hemorrhagic foci (b).

**Figure 4:**
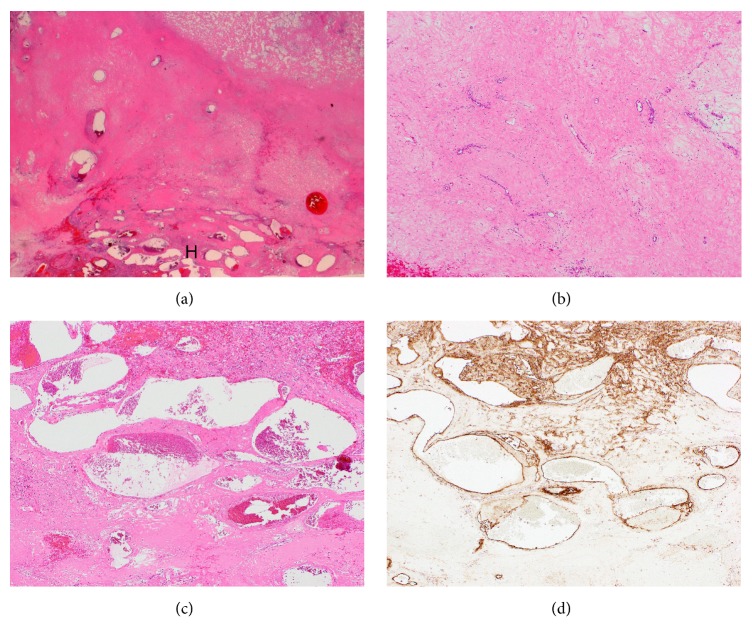
**Histological appearances of sclerosing hemangioma.** (a) Sclerotic area is manly present and cavernous hemangioma area (indicated by H) is partly observed. (Loupe image, HE stain). (b) Sclerotic area presents diffuse fibrosis. (HE stain, x40). (c) Histology of cavernous hemangioma. Note increase and dilation of medium sized veins with cavernous form in b (HE stain, x40). (d) The increased and dilated veins show positivity of CD31 immunostaining being a marker of endothelium in (c) (x40).

The patient underwent posterior sectionectomy. Intraoperative examination revealed a relatively soft dark red tumor (Figure 3(a)); the resected specimen weighed 1.1 kg and measured as 170×100×80 mm. The cut surface of the tumor revealed a white, solid, and cystic mass that was elastic, soft, and homogeneous with a yellowish area considered to be myxoid degeneration (Figure 3(b)). Histological examination showed that the tumor mostly consisted of sclerotic area and cavernous hemangioma area is partly observed (Figure 4(a)). Sclerotic area presents diffuse fibrosis (Figure 4(b)) and the typical histology of cavernous hemangioma was confirmed in some parts. In addition, marked increase and dilation of medium sized veins with cavernous form were frequently noted in the surrounding areas of tumor (Figure 4(c)). The increased and dilated veins show positivity of CD31 immunostaining being a marker of endothelium (Figure 4(d)). The pathologic features were consistent with sclerosing hemangioma. The postoperative course was uneventful, and the patient was discharged on postoperative day 10.

## 3. Discussion

Hepatic sclerosing and sclerosed hemangiomas are very rare benign tumor, but the mechanism responsible for the degenerative changes in hepatic cavernous hemangioma has not been well clarified. Makhlouf and Ishak have reported that there are distinct clinical and histological differences between sclerosing and sclerosed hemangiomas; they suggested that recent hemorrhages and hemosiderin deposits, rich in mast cells are present in sclerosing hemangioma [2]. In the present case, histological examination revealed that the tumor was a sclerosing hemangioma composed mainly of a sclerosed area resulting from changes secondary to ischemic necrosis, venous occlusion by thrombi, and hemorrhage. These features support the contention that sclerosed and sclerosing hemangiomas are fundamentally similar lesions and may represent different stages in the development of the same lesion. From a clinical viewpoint, they also reported that patients with sclerosing hemangioma were younger, and had larger tumors that tended to present as a mass, occurring much more frequently in the right lobe [2]. The clinical features of the present case were well consistent with that report, and we finally diagnosed the lesion as a sclerosing hemangioma on the basis of the histological findings.

Hepatic sclerosing hemangiomas are caused by degenerative changes such as thrombus formation, necrosis, and scar formation within liver cavernous hemangioma, and such varieties of pathological characteristics make precisely radiological diagnosis very difficult [6]. On the other hand, the radiological findings of sclerosing and sclerosed hemangiomas have rarely been reported. In our case, CT showed only marginal enhancement in the peripheral area in the arterial phase and slight internal heterogeneous enhancement in the delayed phase, mimicking adenocarcinoma. MRI showed low intensity on T1-weighted images and some high-signal intensity nodules on T2-weighted images, categorized as non-specific, and not excluding biliary cystadenocarcinoma, mesenchymal tumors with necrosis. Regarding imaging examinations, Yamashita et al. reported that sclerosing hemangiomas exhibit only marginal enhancement on CTHA, whereas the majority of the tumor presents as a perfusion defect [7]. Based on a review of sclerosing and sclerosed hemangiomas, Miyamoto et al. described that MRI revealed a low-intensity signal on T1-weighted images and a high-intensity signal on T2-weighted images [8]. Cheng et al. reported that hyalinized hemangiomas had a signal intensity lower than cerebrospinal fluid on T2-weighted images, lack of early enhancement, and slight peripheral enhancement in the late phase [3]. The collagen-rich and relatively acellular mature fibrous tissue generally has lower signal intensity than muscle on T2-weighted images because of a decreased free water content and a low mobile proton density. Such radiological findings might lead to a preoperative diagnosis of hypovascular adenocarcinoma, including biliary cystadenocarcinoma, cholangiocarcinoma, metastatic liver cancer, mesenchymal tumors, and hepatocellular carcinoma. Preoperatively, abdominal angiography was also performed in this case. To our knowledge, there have been no previous reports that present hepatic angiography image findings of sclerosing hemangioma. This showed a large avascular region in the liver corresponding to the tumor and no typical features of cavernous hemangioma. Ultimately, diagnosis is difficult based on these findings of angiography.

The use of surgical resection for hepatic sclerosing hemangioma is controversial. Most of the tumors reported previously were resected due to preoperative misdiagnosis as hepatic malignancies. Behbahani et al. have shown that knowledge of the appearance of atypical hemangioma and its inclusion in the differential diagnosis of hepatic lesions can alter patient management, being an important aspect to consider before invasive therapies are planned [9]. On the other hand, in fine-needle aspirates, the smears tend to be hemorrhagic, and sometimes only blood is aspirated. Miyamoto et al. have suggested that hepatic resection should be chosen for the management of hepatic sclerosing hemangioma at present [8]. They consider that percutaneous needle biopsy is not acceptable because of the possibility of dissemination of cancer cells if the tumor proves to be malignant.

Including the present case, only 20 cases of hepatic sclerosing hemangioma have been reported in the English literature with detailed information on the patients (Table 1) [3, 4, 7, 10–23] A review of these 20 cases revealed that the average size of the tumor was 86.4 mm, ranging from 8 to 170 mm, and that the mean age of the patients was 63 years, ranging from 39 to 84 years. Our present patient was a very young woman aged 39 years, and the tumor was 170 mm in diameter and weighed 1.1 kg, making this patient the youngest and the tumor the largest to have been reported so far. Of these 20 patients, only 2 (10%) were diagnosed as having sclerosing hemangioma preoperatively.

Sclerosing hemangioma is extremely difficult to differentiate from other hepatic tumors. Further studies in more patients with this tumor are needed to provide an appropriate differential diagnosis of patients of having atypical hemangioma. Therefore, it is critical to be familiar with sclerosing hemangiomas, which leads to preoperative biopsy or intraoperative frozen section to avoid unnecessary extended hepatic resection of this rare benign tumor. However, if tumor malignancy cannot be ruled out in spite of biopsy, hepatic resection should remain the choice for diagnostic surgery at present.

## Figures and Tables

**Table 1:** Cases of hepatic sclerosing hemangioma in the English literature.

Age/sex	Author.	Age/sex	Number of tumor	Size (mm)	CT	MRI (T1/T2)	Preoperaitve diagnosis	Treatment
1986	Takayasu et al.	62F	Solitary	50	Ring E	NA	NA	Surgery
1992	Haratake et al.	65F	Solitary	26	Ring E	NA	Meta/HCC	Surgery
1995	Cheng et al.	NA	Solitary	30	Ring E	Low/Slightly high	Malignant tumor	Surgery
1995	Shim et al.	41F	Solitary	130	Partly filled in	NA	Angiosarcoma	Surgery
2000	Yamashita et al.	67F	Solitary	50	Ring E	High/high	Meta	Surgery
2001	Aibe et al.	67F	Solitary	40	Delayed E	High/high	Meta	Surgery
2005	Lee et al.	65F	Solitary	55	Ring E	Low/moderate	HCC, IHCC, atypical hemangioma	Surgery
2008	Mori et al.	77F	Solitary	95	Ring E	Low/high	IHCC, FLC	Surgery
2008	Choi et al.	63M	Solitary	45	Multifocal patchy E	Low/intermediate	HCC, IHCC, atypical hemangioma	Surgery
2009	Lauder et al.	72M	Solitary	NA	Mild contrast E	NA	Meta	Surgery
2009	Lauder et al.	84M	Solitary	NA	Hypodense	NA	Meta	Surgery
2010	Jin et al.	52M	Solitary	21	Ring E	Low/Slightly high	HCC, Hemangioma	Surgery
2011	Papafragkakis et al.	52F	Solitary	75	Intralesional E	NA	NA	Surgery
2011	Shin YM	50M	Solitary	100	Patch E	Low/high		Obsevation
2012	Yamada et al.	75M	Solitary	8	Ring E	Low/Slightly high	Meta	Surgery
2013	Song et al.	63F	Solitary	91	Ring E	NA	Atypical hemangioma, Meta, HCC	Surgery
2013	Shimada et al.	63M	Solitary	10	Ring E	Low/Slightly high		Surgery
2015	Wakasugi et al.	67F	Multiple	11,28	Ring E	Low/hetero	Meta, HCC	Surgery
2017	Behbahani et al.	70M	Multiple	NA	Ring E	NA		Obsevation
2018	Sugo et al.	39F	Solitary	170	Ring E	Low/Slightly high	Biliary Cystadenocarcinoma	Surgery

E: enhancement, Meta: metastasis, HCC: hepatocellular carcinoma, IHCC: intrahepatic cholangiocarcinoma, FLC: fibromellar HCC.
